# Epidemiology of covid-19 in Mongolia: descriptive findings

**DOI:** 10.1093/eurpub/ckac131.069

**Published:** 2022-10-25

**Authors:** O Amartsengel, D Khishigjargal, N Glushkova, K Lkhagvasuren

**Affiliations:** 1 School of Public Health, Mongolian National University of Medical Science, Ulaanbaatar, Mongolia; 2 Institute of Public Health, Mongolian National University of Medical Science, Ulaanbaatar, Mongolia; 3 Health Information Office, Center for Health Development, Ulaanbaatar, Mongolia; 4 Epidemiology, Biostatistics and Evidence-Based Medicine, Al-Farabi Kazakh National University, Almaty, Kazakhstan

## Abstract

**Background:**

Mongolia is a landlocked country, and has been divided into 21 provinces plus the capital city Ulaanbaatar. In our country, Covid-19 was the first internal case (15 Nov 2020) and the first wave of pandemic occurred (peak at 14 Jun 2021) ten months later on World’s first case and wave. The second and third waves were also delayed by two months, but the fourth wave occurred just in parallel (peak on 17 Jan 2022).

**Methods:**

This study was a retrospective cross-sectional study. We have compared incidence rate (IR) and case-fatality rates (CFR) in provinces by age groups based on publicly available data reported by MoH from January to December of 2021.

**Results:**

CFR in Mongolia was low (average 0.23%), and had a clear dynamic to drop from beginning to the present time (for I wave-0.49, and II-0.42, III-0.23, IV-0.04 correspondently). At the beginning of the III waves, we vaccinated 68.7% of the total population, and in the fourth wave, CFR significantly decreased. IR had two peaks: in the age group 30-34 (250.6) and over 85 (248.9). There was a strong correlation between age and morbidity. Up to age group 40-44 (0.12 %), there was a deliberate increase of CFR, further up to 65-69 ages (2.7%) gradual increase, and from age group 70-74 CFR rapidly increases reaching 10.5% at age group over 85. IR was higher in men aged 65 years and over. CFR was significantly higher in Ulaanbaatar city and Umnugovi province morbidity due to Covid-19 not being registered.

**Conclusions:**

Vaccination in Mongolia was a key factor in declining fatality. Umnugovi province is crucial for exports to China, so we suppose, government decisions strongly affected in fatality rate in this province, in terms of the evacuation of severely diseased patients to the capital.

**Key messages:**

• Vaccination is played important role in COVID-19 epidemiology.

• Burden of Covid-19 need to be investigated deeply in association with political decisions.

